# Acupuncture-Induced Tension Pneumothorax Presenting as Acute Heart Failure

**DOI:** 10.1155/2021/9986300

**Published:** 2021-10-05

**Authors:** Alia Arif Hussain, Jeppe Nygaard, Kasper Kofod Pedersen, Celi Anne Schoenike, Erik Kovacs, Steen Hylgaard Jørgensen

**Affiliations:** ^1^Emergency Department, North Denmark Regional Hospital, Denmark; ^2^Department of Abdominal Surgery, North Denmark Regional Hospital, Denmark; ^3^Department of Radiology, North Denmark Regional Hospital, Denmark; ^4^Department of Cardiology, North Denmark Regional Hospital, Denmark

## Abstract

Takotsubo syndrome (TSS) is a reversible, acute cardiomyopathy with transient heart failure, often secondary to other disorders. A 64-year-old woman, with no history of ischemic heart disease, was admitted to the emergency department after developing sudden-onset dyspnea after a planned acupuncture treatment for back pain. Acute echocardiography showed decreased left ventricular function with basal hypercontraction and apical akinesia and was interpreted, and treated, as acute heart failure. When the attending cardiologist arrived, the patient still had dyspnea with a declining blood pressure (97/65 mmHg) and tachycardia (111/minute). The cardiologist suspected a tension pneumothorax induced by the penetration of an acupuncture needle to the apex of the lung, as well as secondary TSS cardiomyopathy. An acute chest X-ray was performed, which showed a large left-sided rim pneumothorax. The attending surgeon placed a chest tube in the left 6th intercostal space in the midaxillary line, and the patient reported immediate pain relief and improvement in her dyspnea. The patient's clinical condition improved, and a control X-ray showed that the lung was fully expanded. The chest tube was removed, but after a few minutes, the patient developed a massive subcutaneous emphysema in the upper chest and in the face and her clinical condition deteriorated rapidly. A new chest tube was inserted, and the patient's tachycardia diminished, with her clinical condition improving immediately. The patient remained hospitalized for the next seven days. After three continuous days without any escaped air in the chest tube, the tube was removed, and the patient was observed for another 48 hours. This time, the removal was without any complications and within two days, the patient was ready for discharge. The follow-up echocardiography showed complete recovery of left ventricular function.

## 1. Introduction

Takotsubo syndrome (TSS) is a reversible, acute cardiomyopathy with transient heart failure, often secondary to other disorders [1, 2]. The word “takotsubo” is Japanese and translates to “octopus trap”, because when affected by TSS, the left ventricle of the heart takes on a shape resembling an octopus trap. Recently, a systematic review and meta-analysis reported that 36% of patients had experienced physical stress prior to TSS. Chest pain and dyspnea were reported in an additional 64% and 26%, respectively [3]. Acupuncture is associated with rare but serious complications including pneumothorax, subarachnoid hemorrhage and cardiac tamponade [4–6]. This case report describes a case of acupuncture-triggered tension pneumothorax with secondary takotsubo.

## 2. Case Report

**Figure 1:**
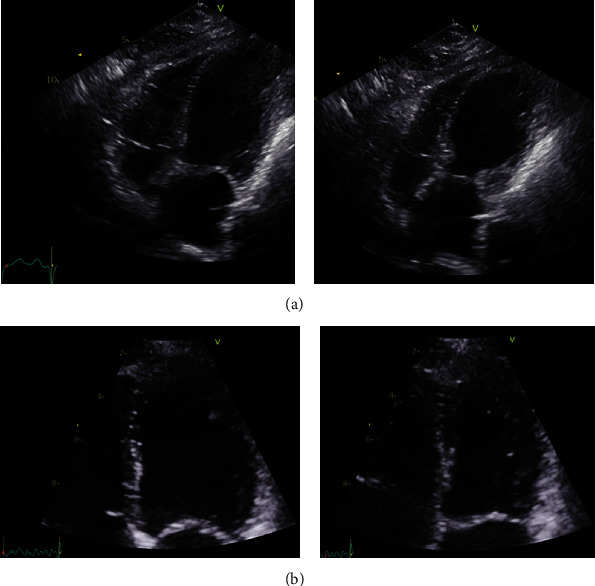
(a) Echocardiography in the acute phase. The first echocardiography showing modified 4 ch view of end-diastolic and end-systolic left ventricular volume. Basal hypercontraction and apical ballooning is evident, interpreted as acute heart failure/takotsubo cardiomyopathy. (b) Echocardiography after remission of tension pneumothorax and subcutaneous emphysema. Follow-up echocardiography showing 4 ch view of end-diastolic and end-systolic left ventricular volume 1 month later. Left ventricular ejection fraction (LVEF) has normalized.

**Figure 2:**
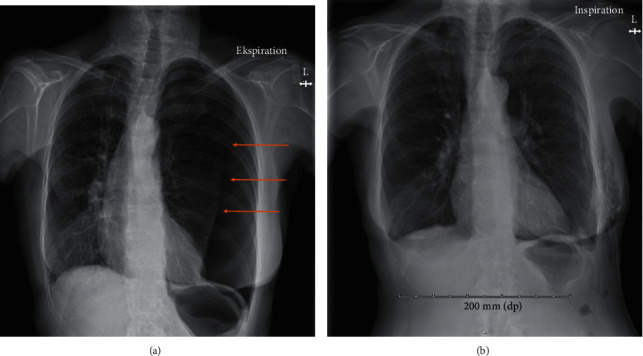
(a) Chest X-ray showing tension pneumothorax of the left lung. The first chest X-ray showed a large left-sided rim pneumothorax, extending along the entire lateral border of the lung, from the base to the apex, and with a maximal width of 6 cm at the base of the left lung. (b) Chest X-ray showing subcutaneous emphysema. A postintervention chest X-ray showed an intercostal drain placed at about the 8th intercostal space on the left side. A near complete regression of the pneumothorax is seen, with the lung parenchyma again extending to the borders the left hemithorax and a smaller residual rim apicolaterally measuring 12 mm. In the soft tissues at the puncture point, multiple diffuse hypodense areas are seen, resembling subcutaneous emphysema.

A 64-year-old woman, with a history of mild chronic obstructive pulmonary disease (COPD) and with no history of ischemic heart disease, was admitted to the emergency department (ED) at North Denmark Regional Hospital. The patient reported severe, sudden-onset shortness of breath and chest pain, starting ten minutes after the patient had left her general practitioner for a planned acupuncture appointment. In the ambulance, the patient was treated with inhalations of salbutamol, which reduced her chest pain. Sublingual administration of nitroglycerin and aspirin did not further ameliorate symptoms. Upon arrival to the ED, the patient had dyspnea while speaking and was sitting up in bed, using axillary respiratory muscles; peripheral oxygen saturation (SpO_2_) was 80% without oxygen therapy; respiratory rate was 25/minute; heart rate (HR) was 127 bpm; and blood pressure was 116/83 mmHg. The SpO_2_ increased to 96% with nasal oxygen therapy set to 10 liters/minute. Auscultation of the lungs revealed vesicular respiration with normal breath sounds, possibly with slightly decreased breath sounds over the base of the left lung. Chest-wall palpation did not evoke pain nor did the patient have leg edema. The ECG showed sinus tachycardia without signs of ischemia, and blood tests were normal. The arterial blood gas (ABG) indicated a metabolic acidosis without respiratory compensation (pH 7.27, pO_2_ 7.8 kPa and pCO_2_ 5.5 kPa) with elevated lactate of 3.7 mmol/L. An echocardiography (Figure 1(a)) performed in the ED showed decreased left ventricular ejection fraction (LVEF) with basal hypercontraction and apical akinesia, which was interpreted as acute heart failure. The patient was transferred to an in-patient department with the suspicion of a cardiac cause to her symptoms. Thus, the attending cardiologist was summoned. When the cardiologist arrived, the patient still had dyspnea with a declining blood pressure (97/65 mmHg) and tachycardia (111/minute). Auscultation of the lungs revealed reduced breath sounds on the left side with hyperresonance/hypertympanism over the left hemithorax as compared to the right. The cardiologist suspected a tension pneumothorax induced by the penetration of an acupuncture needle to the apex of the lung, as well as secondary TSS cardiomyopathy. The patient's blood pressure remained low but unchanged, and therefore, an acute chest X-ray was performed (Figure 2(a)), which showed a large left-sided rim pneumothorax, extending along the entire lateral border of the lung from the base to the apex. The attending surgeon placed a 24-French chest tube left at the 6th intercostal space in the midaxillary line. The patient reported immediate pain relief and improvement in her dyspnea. A chest X-ray confirmed that the chest tube was placed correctly. The patient was transferred to the general surgery ward for observation and pain management. Two days later, the patient's clinical condition had improved, and a control X-ray showed that the lung was fully expanded except for a small 1 cm section in the apical part of the left pleural cavity. The chest tube was removed, but after a few minutes, the patient developed a massive subcutaneous emphysema in the upper chest and in the face and her clinical condition deteriorated rapidly. The patient had tachycardia and dyspnea and was unable to open her eyes due to the massive subcutaneous emphysema (Figure 2(b)). A new chest tube was inserted, and the patient's tachycardia diminished, with her clinical condition that improved immediately. Seven days later, no air was seen escaping via the chest tube and a chest X-ray showed an improvement in the subcutaneous emphysema with only a small pneumothorax (1 cm) remaining in the apical part of the lung. Once again, the drain was removed, and four hours later, the patient again developed dyspnea and massive subcutaneous emphysema with a pulse rate of 150 bpm, blood pressure of 172/97 mmHg, and an SpO_2_ of 97% on 15 liters of oxygen/minute. A new chest tube was inserted, and the effect was immediate, as made apparent by reduction in the patient's dyspnea, as well as a stabilization of her vital signs.

The patient remained hospitalized for the next seven days. Thoracic surgeons were consulted, but they found no evidence of pleural bullae on the chest CT. After three continuous days without any escaped air in the chest tube, the tube was removed, and the patient was observed for another 48 hours. This time, the removal was without any complications and within two days, the patient was ready for discharge. The follow-up echocardiography showed complete recovery of left ventricular function (Figure 1(b)).

## 3. Discussion

In the presented case, a 64-year-old woman, with no history of ischemic heart disease, developed severe sudden-onset dyspnea after a planned acupuncture treatment for back pain by her general practitioner. Echocardiography showed decreased LVEF with basal hypercontraction and apical akinesia and was interpreted as acute heart failure. When the attending cardiologist arrived, the patient's blood pressure had decreased (to 97/65 mmHg), and auscultation of the lungs revealed reduced breath sounds on the left side. Tension pneumothorax was suspected and confirmed by an acute chest X-ray. The tension pneumothorax was presumably induced by an acupuncture needle penetrating the apex of the lung, resulting in subsequent secondary takotsubo cardiomyopathy. In the present case, we had no information about the general practitioner's experience with acupuncture.

TSS, also known as stress cardiomyopathy, is characterized by a temporary weakening of the left ventricle most probably caused by an increase in catecholamine concentration triggered by significant physical stress.

Tension pneumothorax is a rare but serious and potentially fatal complication of acupuncture. A history of thoracic surgery, chronic bronchitis, emphysema, pneumonia, tuberculosis, and lung cancer may increase the postacupuncture risk of iatrogenic pneumothorax [7]. In the present case report, TSS temporarily concealed the underlying tension pneumothorax. However, TSS was secondary to the physical stress of the tension pneumothorax but due to fast, bedside ultrasound diagnostics, the patient's symptoms were treated assuming a cardiac origin.

To our knowledge, this is the first reported case of TSS secondary to an acupuncture-induced tension pneumothorax [8–12]. This case illustrates that TSS is often caused by a physical or emotional stressor, which should be diagnosed and treated immediately. Furthermore, both the patient and acupuncturist should be informed and aware of complications related to acupuncture in the thoracic region, especially the possibility of injuries to internal organs and the early symptoms of pneumothorax. In high-risk patients, an observation period of 15 minutes after acupuncture in the thoracic region could ensure earlier detection of fatal complications such as tension pneumothorax.

## Data Availability

Additional data used to support the findings of this study are available from the corresponding author upon request.
